# Outer Membrane Vesicles (OMVs) of Gram-negative Bacteria: A Perspective Update

**DOI:** 10.3389/fmicb.2017.01053

**Published:** 2017-06-09

**Authors:** Arif Tasleem Jan

**Affiliations:** Department of Medical Biotechnology, Yeungnam UniversityGyeongsan, South Korea

**Keywords:** Gram-negative bacteria, immune system, Outer Membrane Vesicles (OMVs), pathogenesis, vaccines

## Abstract

Outer Membrane Vesicles (OMVs) of Gram-negative bacteria are spherical membrane-enclosed entities of endocytic origin. Reported in the consortia of different bacterial species, production of OMVs into extracellular milieu seems essential for their survival. Enriched with bioactive proteins, toxins, and virulence factors, OMVs play a critical role in the bacteria-bacteria and bacteria-host interactions. Emergence of OMVs as distinct cellular entities helps bacteria in adaptating to diverse niches, in competing with other bacteria to protect members of producer species and more importantly play a crucial role in host-pathogen interaction. Composition of OMV, their ability to modulate host immune response, along with coordinated secretion of bacterial effector proteins, endows them with the armory, which can withstand hostile environments. Study of the OMV production under natural and diverse stress conditions has broadened the horizons, and also opened new frontiers in delineating the molecular machinery involved in disease pathogenesis. Playing diverse biological and pathophysiological functions, OMVs hold a great promise in enabling resurgence of bacterial diseases, in concomitance with the steep decline in the efficiency of antibiotics. Having multifaceted role, their emergence as a causative agent for a series of infectious diseases increases the probability for their exploitation in the development of effective diagnostic tools and as vaccines against diverse pathogenic species of Gram-negative origin.

## Introduction

The era of existence of prokaryotes, as autonomous structures has prevailed for a long time. In comparison with the the individual survival traits with few interactions and no compartmentalization (Manning and Kuehn, 2013), a different group of bacteria has emerged with survival strategies similar to the eukaryotes (Raymond and Bonsall, 2013; Spitzer and Poolman, 2013). With ordered structural organization, fine-tuned physiology, and interactive social behavior, a paradigm shift for better survival is observed in bacteria. Of the differently adapted modes, production of Outer Membrane Vesicles (OMVs) by Gram-negative bacteria, plays a prominent role in interaction among themselves or with the host (Bonnington and Kuehn, 2014; Haurat et al., 2015).

Outer Membrane Vesicles are enriched with proteins that enhance their invasive abilities, thereby promoting efficient internalization of OMVs at the host interface. The best examples include, outer membrane (OM) localized invasins, IpaB, IpaC, and IpaD of *Shigella flexneri* and Ail protein of *Escherichia coli* (Kuehn and Kesty, 2005). OM proteins OspA and OspB attribute adhesive properties to OMVs, and thus enhance binding ability of bacteria to host cell receptors (Shoberg and Thomas, 1993). After adherence to host cells, evasion of the host defense system, or the modulation of the host immune responses, is triggered due to the secretion of toxins such as shiga toxin (Stx1 and Stx2), vacuolating toxin (VacA), heat labile toxin (LT), cholera toxin (CT), etc., and virulence factors such as proteases, glycoproteins, etc. (Ellis and Kuehn, 2010; Chattopadhyay and Jaganandham, 2015). Summary of bacterial OMV production, their properties and functional roles, in bacteria-bacteria and host-bacteria interactions, have been provided in **Table 1**.

**Table 1:** Bacterial species showing OMV production.

S. No	Bacterial species	Virulence factors as OMV component	Associated function	Reference
1	*Escherichia coli* [Enterotoxigenic *E. coli* (ETEC), Shiga toxin producing *E. coli* (STEC), Enterohemorrhagic *E. coli* (EHEC)]	Heat labile enterotoxin (LT), Shiga toxin, Cytolysin A (ClyA)	Pore forming ability, enterotoxic and vacuolating activity, cytotoxicity	Kolling and Matthews, 1999; Horstman and Kuehn, 2000; Yokoyama et al., 2000; Wai et al., 2003; Kuehn and Kesty, 2005; Kwon et al., 2009; Mendez et al., 2012; Jun et al., 2013
2	*Helicobacter pylori*	Vacuolating toxin (VacA), Lewis antigen LPS, Helicobacter cysteine rich proteins (Hcp), Sialic acid binding adhesion (SabA)	Adherence, cytotoxic and vacuolating activity, cell proliferation activity	Fiocca et al., 1999; Keenan et al., 2000; Mullaney et al., 2009; Olofsson et al., 2010; Jun et al., 2013
3	*Pseudomonas aeruginosa*	Alkaline phosphatase, Phospholipase C Protease, Hemolysin, Pseudomonas quinolone signal (PQS), Cif, hydrolases	*In vitro* enzyme activities, cytokine stimulation, bactericidal quinolines	Kadurugamuwa and Beveridge, 1995, 1996; Li et al., 1998; Mashburn and Whiteley, 2005; Mashburn-Warren et al., 2008; Bomberger et al., 2009; Ellis et al., 2010; Choi et al., 2011; Toyofuku et al., 2012
4	*Borrelia burgdorferi*	Outer surface proteins (OspA, B, D)	Adherence to host cells	Dorward et al., 1991; Shoberg and Thomas, 1993, 1995
5	*Shigella flexneri*	Invasion plasmid antigens (IpaB, C,D)	Invasion of host tissue	Kadurugamuwa and Beveridge, 1998
6	*Shigella dysenteriae*	Shiga toxin (Stx)	Cytotoxicity, host cell apoptosis	Dutta et al., 2004
7	*Salmonella typhi*	Outer membrane protein (OmpC), ClyA	Pore forming activity	Bergman et al., 2005
8	*Treponema denticola*	Proteases, Dentilisin	Chymotryptic activity, disruption of tight junctions	Rosen et al., 1995; Chi et al., 2003
9	*Neisseria meningitis*	NarE, NlpB, PorA, B	Cytokine production, fibrinolytic activity, adherence to host cells	Ferrari et al., 2006; Vipond et al., 2006; Massari et al., 2010; Van De Waterbeemd et al., 2013
10	*Bordetella pertussis*	Pertussis toxin (Ptx), Adenylate cyclase hemolysin	Cytotoxicity	Hozbor et al., 1999
11	*Burkholderia cepacia*	Phospholipas-N, Hemagglutinin	Enzyme activities	Allan et al., 2003
12	*Vibrio cholera*	Rtx toxin, LPS	Depolymerising actin, stimulatory response	Bishop et al., 2010; Altindis et al., 2014
13	*Xanthomonas campestris*	Type-3 secretion proteins, cellulase, xylosidae	Enzyme activity, insecticidal activity	Sidhu et al., 2008
14	*Legionella pneumophila*	Acid phosphatase (Map), Protease (Msp), Chitinase (ChiA), Hsp60	Adherence to ECM, enzyme activity	Fernandez-Moreira et al., 2006; Galka et al., 2008
15	*Moraxella catarrhalis*	Ubiquitous surface protein (UspA1, A2)	Complement binding	Tan et al., 2007; Vidakovics et al., 2010
16	*Acinetobacter baumannii*	Outer membrane protein (AbOmpA), PAMPS (LPS, flagellin),Proteases, Phospholipases, SOD, Catalase	Binding to host tissues, Immunomodulatory effect, enzyme activity	Kwon et al., 2009; Mendez et al., 2012; Moon et al., 2012; Jun et al., 2013
17	*Campylobacter jejuni*	Cytolethal distending toxin (CDT)	Adhesion and invasion, immunomodulatory effect	Elmi et al., 2012; Jang et al., 2014
18	*Porphyromonas gingivalis*	CTD family proteins such as gingipains (RgpA, RgpB, Kgp)	Adherence, host tissue Invasion, immune evasion	Veith et al., 2014
19	*Yersinia pestis*	Adhesin Ail, Protease Pla, F1 outer fimbrial antigen	Complement binding, enzyme activity	Eddy et al., 2014
20	*Cronobacter sp. [C. sakazakii, C. turicensis, C. malonaticus]*	Outer membrane protein (OmpA and OmpX)	Binding to host cell receptors	Kothary et al., 2017


In addition to their role in interspecific competition, release of OMVs greatly enhances the survival scenario for a bacterium by subduing effects of bacteriophages and antimicrobial peptides, during the combat (Manning and Kuehn, 2011). They also have a role as a signaling molecule, rendering help by abetting inter- and intra-species communication. Despite this, exosome production, loading of cellular components as cargo, conditions that promote their formation and the role it plays under different circumstances, is yet not clearly established. Hitherto, a lacuna in our understanding of the molecular mechanisms for OMV formation, hampers to ascertain their physiological relevance *in vivo*. In this review, multifaceted aspects of OMV production – their biogenesis, cargo selection and secretory mechanisms, physiological and pathological functions – with respect to the progression of diseases, and possibility of exploiting OMVs in the development of effective diagnostic tools, have been discussed. It is anticipated that the study will broaden our understanding of the OMV biology and will confer new avenues in the use of OMVs for diverse biotechnological applications.

## Outer Membrane Vesicles

Outer Membrane Vesicles are small, spherically bilayered (100–300 nm) vesicles released into extracellular milieu from the OM of Gram-negative bacteria (Beveridge, 1999). Several bacterial species have been reported to produce OMVs, such as *Escherichia coli* (McBroom and Kuehn, 2007; Schwechheimer and Kuehn, 2015), *Pseudomonas aeruginosa* (Bauman and Kuehn, 2006), *Shigella* sp. (Kadurugamuwa and Beveridge, 1999), *Salmonella* sp. (Elhenawy et al., 2016), *Helicobacter pylori* (Fiocca et al., 1999; Turner et al., 2015), *Campylobacter jejuni* (Lindmark et al., 2009; Elmi et al., 2012), *Borrelia burgdorferi* (Dorward et al., 1991; Shoberg and Thomas, 1993), *Vibrio* sp. (Chatterjee and Chaudhuri, 2011), and *Neisseria* sp. (Devoe and Gilchrist, 1973; Pettit and Judd, 1992) They carry lipopolysaccharides (LPS), phospholipids, peptidoglycan, outer membrane proteins (OMPs), cell wall components, proteins (periplasmic, cytoplasmic, and membrane-bound), nucleic acids (DNA, RNA), ion metabolites and signaling molecules as cargo to them (Lindmark et al., 2009; Koeppen et al., 2016; Vanaja et al., 2016). Owing to the nature of cargo transference, they are anticipated to play a role in the bacterial adherence with the host. They are also involved in stress responses, which involves biofilm formation, inter- and intraspecies delivery of molecules, resistance against antibiotics and modulation of the host immune response.

The ability of OMVs to transfer biological molecules to the host cell, makes their production purposeful among Gram-negative bacteria. Apart from their role in bacterial communication, transfer of virulence factors as cargoes to OMVs, enhances bacterial survival inside the host (Ellis and Kuehn, 2010; Chattopadhyay and Jaganandham, 2015). The development of secretory systems (I – VI) in Gram-negative bacteria has helped in secretion of virulence factors across the bacterial envelope into the extracellular space. Secreted as distinct entities, OMV mediated transfer of virulence factors, adhesion molecules, toxins and other immunomodulatory compounds, constitutes a separate secretory system, that operates in Gram-negative bacteria to gain access to host tissues and bloodstream.

## Production of OMVs

Production of OMVs under *in vitro* conditions has been reported during the bacterial growth on solid and in liquid media, in biofilms (Schooling and Beveridge, 2006; Klimentova and Stulik, 2015), and also during intracellular infections (Namork and Brandtzaeg, 2002; Unal et al., 2011). As growth conditions have significant influence on the vesiculation process, late grown cells display maximum OMV yields (Klimentova and Stulik, 2015). However, cell death which unavoidably happens during the later stages of the bacterial growth, causes contamination with membrane components and cytosolic proteins. In addition, nutrient unavailability and increased waste disposal at later stages of progressive growth, affects composition profile of OMVs both qualitatively and quantitatively (Tashiro et al., 2010; McCaig et al., 2013; Klimentova and Stulik, 2015). Representing a mechanism to alleviate stress, factors such as temperature, nutrient depletion and exposure to antibiotics, increases packaging and release of the materials (Collins, 2011; Klimentova and Stulik, 2015). Given the fact that inside ambience of host cells remains harsh, host-pathogen interactions itself modifies the composition and production of OMVs (Kuehn and Kesty, 2005). In short, changes in the dynamics of OMVs, with regard to the varying nature of its contents during different growth stages, affects their fate and biological functioning.

## Biogenesis of OMVs

**FIGURE 1:**
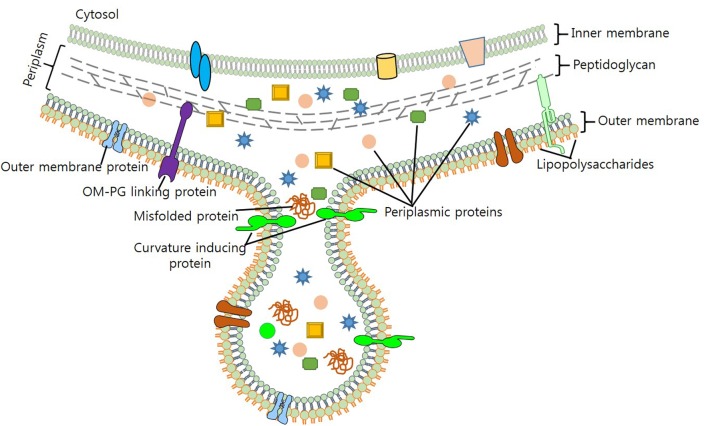
Biogenesis of OMV production in bacteria. Figure depicts the composition of OMV, cargo selection and loading as part of OMVs.

Maintaining viability during vesicle formation makes process of vesiculation complex and elusive. In light of the evidence generated through various biochemical and genetic studies, several models for the mechanistic production of OMVS have been put forth. Previous studies – by Burdett and Murray (1974) and Hoekstra et al. (1976) – on the biogenesis of OMVs suggest that reduction in the cross-linking between peptidoglycan (PG) and OM triggers their formation (Burdett and Murray, 1974; Hoekstra et al., 1976). As lipoprotein Lpp contributes to OM-PG linkage, it was hypothesized, that hypervesiculation could arise due to a mutation in the *lpp* gene (Wensink and Witholt, 1981; Bernadac et al., 1998; Cascales et al., 2002). *vfgl*, a different lipoprotein involved in the synthesis and degradation of PG, is associated with OMV production in *E. coli* (AIEC) and *E. coli* K12 strain (Rolhion et al., 2005). Contribution of *vfgl* in OMV production is presumably linked with the increase of PG production or via down-regulation of lytic transglycosylases, which are associated with the maintenance of turgor pressure on OM (Eggert et al., 2001; Rolhion et al., 2005). Increase in the number of OMVs produced as blebs to OM (**Figure 1**), relieves cell of the turgor pressure exerted by PG and muramic acid during cell wall synthesis. Although biochemical screening has revealed that OMVs and OM share a similar protein profile, yet, it remains unclear, how cytosolic molecules are packaged to OMVs.

Mashburn and Whiteley (2005) proposed that enrichment of OM with phospholipids, LPS and other specific molecules, brings curvature changes in OM that leads to OMV production (Mashburn and Whiteley, 2005). Work done on *P. aeruginosa* unveils membrane curvature transformations brought by the membrane insertion of a quorum sensing molecule PQS (2-heptyl-3-hydroxy-4-quinolone), results in OMV production (Mashburn-Warren et al., 2009; Schertzer and Whiteley, 2012). Sequestration of positively charged compounds and destabilization of Mg^2+^ and Ca^2+^ salt bridges by PQS, increases anionic repulsion of LPS molecules; thereby increasing OMV production (Mashburn and Whiteley, 2005). Enhanced OMV production has been observed by the addition of chelating agents (EDTA), however, addition of Mg^2+^ to *P. aeruginosa* results in the antagonist effect (Mashburn and Whiteley, 2005; Lee et al., 2016). On the contrary, proteins of OM such as OmpA, TolA/B (Tol-Pal), YbgF, and LppAB – which stabilize OM by enhancing protein-protein or protein-PG interactions – also contribute to the biogenesis of OMVs (Schwechheimer et al., 2013). Moreover, stress such as high temperature, presence of contaminants such as antibiotics also increases OMV production (Kulp and Kuehn, 2010; MacDonald and Kuehn, 2012).

## Composition of OMVs

Analysis of OMVs, purified by density gradient centrifugation revealed their constituents i-e. proteins and lipids of OM, periplasm, along with different cytoplasmic components. Regarded as distinct cellular entities, following section summarizes the information about the constituents of OMVs, which includes proteins, lipids and other entities:

### Proteins

Study of OMVs reveal abundance of OM proteins (OMPs; OmpA, OmpC, and OmpF), periplasmic proteins (AcrA and alkaline phosphatase) and a series of virulence factors involved in the adhesion and invasion of host tissues. With advancements in the MS-based proteomic profiling technologies, identification of more than 3500 proteins – belonging to diverse functional categories – became possible (Kim et al., 2013). Evidence suggests that protein cellular localization greatly affects its inclusion to OMVs, as observed for *H. pylori* and *Serratia marcescens* (Olofsson et al., 2010; McMahon et al., 2012). Periplasmic proteins associated with the inner leaflet of OM exhibit increased incorporation within OMVs, in comparison with the proteins that are tightly bound to inner membrane. Although majority of OMPs from *H. pylori* load as cargo to OMVs, an anomalous behavior in cargo loading behavior in *S. marcescens* is also observed (Olofsson et al., 2010; McMahon et al., 2012). In *S. marcescens*, proteins such as Omps, maltoporin, and TolC, that show abundance on OM, goes undetected in OMVs. Failure in export of MipA in *S. marcescens*, as a part of OMVs and vice versa, goes unnoticed in the OM (McMahon et al., 2012). Although, actual mechanism for this mysterious behavior remains elusive, the decision of secretion or cellular retention is believed to occur via a series of protein and lipid recruitment factors. Contrastingly, shiga toxin of *S. dysenteriae* promotes its secretion by enhancing OMV production (Yokoyama et al., 2000). Upholding strain-specific characteristics, preferential packaging of proteins to OMVs is influenced by protein content of OMVs. As packaging process depends on the concentration ratio of OMV with its cellular contents, enrichment of proteins to OMVs occurs when protein contents of OMVs, that are normalized to OM, are significantly higher with respect to their cellular concentration (Bonnington and Kuehn, 2014).

### Lipids

Lipids represent important structural components of Gram-negative OMVs. Sharing similarity with OM (Chowdhury and Jagannadham, 2013; Kulkarni and Jagannadham, 2014), there are reports of some lipids being present in OMVs but not as part of OM (Kato et al., 2002). Horstman and Kuehn (2000) reported that enterotoxigenic *E. coli* OMVs have glycerophospholipids, phosphatidylglycerol, phosphatidylethanolamine, and cardiolipin as its major lipids, which are associated with the curvature of OMVs (Horstman and Kuehn, 2000). Chowdhury and Jagannadham, (2013) revealed fine structural characterization of OMVs while performing mass spectrometric (MS) studies on *P. syringae* (Chowdhury and Jagannadham, 2013). They found, phosphatidylglycerol and phosphatidylethanolamine are the major lipid components in the OMVs. Similarly, other studies also reveal that phosphatidylglycerols are major constituents in OMVs, however, it was also seen that phosphatidylethanolamines compose the major portion of OM (Tashiro et al., 2011). Moreover, higher proportion of saturated fatty acids in OMVs attributes them with a rigid structure.

Performing functions of adhesion in biofilms, LPS (characteristic of Gram negative OM) are also present as component to OMVs. Similar to proteins, only a fractional amount of parent LPS are present in the OMVs (Kulkarni and Jagannadham, 2014). Expressing two types of O-antigen side chains, *P. aeruginosa* show enrichment of negatively charged B-band forms of LPS in OMVs, compared to neutral A-band (Kadurugamuwa and Beveridge, 1995). Enrichment of OMVs with B-band LPS is believed to be the consequence of the charge repulsive forces between adjacent B-band molecules in OM. Contrastingly, enrichment with A-band LPS of OMVs has been observed in the dental pathogen, *Porphyromonas gingivalis* (Haurat et al., 2011).

### Nucleic Acids

Outer Membrane Vesicles carry both luminal and surface associated DNA. A clear distinction between them arises on DNase treatment; luminal DNA being resistant persists even after the treatment process (Renelli et al., 2004). With this, several different forms of luminal DNA have been reported from *E. coli*, *N. gonorrhoeae*, *P. aeruginosa*, and *H. influenza* (Dorward and Garon, 1989; Kolling and Matthews, 1999; Mashburn-Warren and Whiteley, 2006; Lee et al., 2008). In addition to DNA, RNA, plasmid and DNA of phage and chromosomal DNA, has also been reported in OMVs. Although actual mechanisms of nucleic acid incorporation remain unclear, but it is believed that nucleic acid incorporation into the interior of OMVs happens from the lysed remains of cells that are present in the milieu, during the process of biogenesis (Yaron et al., 2000; Renelli et al., 2004).

Sorting of individual cellular components and their loading as cargo, controls composition and distribution of OMVs, and as such their specificity in counteracting the immune defense system of host. Acting as a signature to bacterial fitness, characteristic enrichment of proteins, lipids and nucleic acids to lumen or localization to OMV membrane, sheds light on the physiological functioning of OMVs and their association in increasing the bacterial survivability in their niche.

## Function of OMVs

**FIGURE 2:**
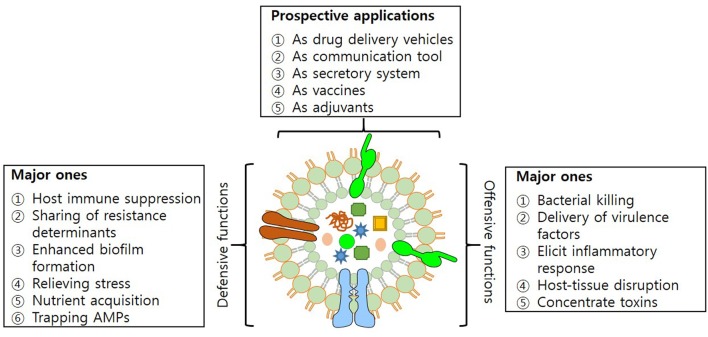
Structure of OMV. Figure illustrating offensive and defensive roles of OMVs utilized in bacteria-bacteria and bacteria-host interactions; and their potential applications.

Outer Membrane Vesicles have characteristics that enable them to mediate transfer of DNA fragments, autolysins, cytotoxins, virulence factors and a variety of other biomolecules (Alaniz et al., 2007; Furuta et al., 2009; Biller et al., 2014; Fulsundar et al., 2014). Their secretion helps bacteria in establishing inter- and intra-species communication and also strengthens the interaction with the host. Among the prominent roles in diverse physiological and pathological functions, OMVs have been recognized for their role in acquisition of nutrients, stress responses and delivery of toxins, adhesion and virulence factors to evade host defense system (**Figure 2**).

### Bacterial Mortality and Nutrient Acquisition

Differences in peptidoglycan composition makes bacteria prone to death by OMVs. The killing effect is prominent for bacterial types that show similarity in peptidoglycan composition, as that of OMV donors (Kulkarni and Jagannadham, 2014). Neutralization of bacteria is compromised due to the presence of similar degradative enzymes in OMVs and bacteria, making bacteria less susceptible to degradation. Moreover, fusion of OMVs with a non-self-strain increases their susceptibility toward degradative enzyme machinery (Beveridge, 1999). The enzyme cargo of OMVs administers bacteria with the capability of making distinction between self and non-self populations, enabling target specific killing of non-self-bacteria (Vasilyeva et al., 2008). An excellent example of this system is operational in the *Lysobacter* sp. – that secretes endopeptidase L5 – which is capable of degrading competing Gram-negative bacteria (Vasilyeva et al., 2008). A similar mechanism also operates for PG hydrolases. OMVs that possess PG hydrolases produce extermination effects after making a clear distinction for non-self-bacteria (Kadurugamuwa and Beveridge, 1996; MacDonald and Kuehn, 2012).

Of the versatile roles, packaging of enzymes such as proteases and glycosidases as cargoes to OMVs, plays a prominent role in the acquisition of nutrients for bacterial communities. OMV associated DNA and proteins function as a source of carbon and nitrogen during the bacterial growth. OMVs of *Myxococcus xanthus* carry alkaline phosphatase, which upon act on competitive bacteria cause release of phosphate that promotes development of multicellular community (Evans et al., 2012; Berleman et al., 2014). Phosphoenolpyruvate, a catalytic product of OMVs carrying enolase, converts plasminogen to plasmin. Phosphoenolpyruvate also aids in the bacterial colonization of the host, following the degradation of matrix proteins (Toledo et al., 2012). The scarcity of metal ions in the bacterial habitats leads to inter- and intra-species competition (Kulp and Kuehn, 2010). In addition to serving as an arsenal in interspecies competition, enrichment of OMVs with rare metal ions makes them available for easy disposal to microbial use.

### Stress Response and Biofilm Formation

Mutation in the stress responsive genes increases production of OMVs in the bacteria. Exposure of cells to environmental contaminants (antibiotics) has potentially evolved bacterial OMVs, either with multidrug efflux pumps capabilities or with ability to catalyze degradation by sequestering antibiotics from the extracellular milieu (Ciofu et al., 2000; Manning and Kuehn, 2011). Acting as nutrient sensors and transporters of essential molecules, enhanced expression of surface receptors and ABC transporter in OMVs increases bacterial survival. The release of unfolded and misfolded proteins to OMVs gives an impression of its development, by sensing a stress responsive state (Baumgarten et al., 2012).

Biofilms are surface adhering structures produced in response to stress by bacteria (Kulp and Kuehn, 2010; Manning and Kuehn, 2011). Matrix assisted biofilms contain polysaccharides, lipids, nucleic acids and protein entities like flagella, pili and OMVs. OMVs mediate delivery of growth factors and components of the extracellular matrix (Schooling and Beveridge, 2006; Klimentova and Stulik, 2015). Release of exopolysaccharides through OMVs increases co-aggregation of cells in the biofilms. Shift from free living to the sedentary state of the bacterial population in biofilms protects cells from desiccation, starvation and adverse effects of antimicrobial agents (Klimentova and Stulik, 2015). Production of OMV helps bacteria to overcome the effects of antimicrobial peptides. Acting as carriage of resistant determinants like β-lactams, and enzymes such as protease, endopeptidases, etc., OMVs give survival advantage to bacteria due to antibiotic resistance traits via biofilms, thereby protects bacteria from antibiotic carnage (Beveridge et al., 1997). Represented as decoy entities, vesicle associated multidrug efflux pumps contribute in transient survival of susceptible bacteria in their surroundings. Association of OMVs with the biofilms of *P. aeruginosa* has been already reported (Beveridge et al. (1997), subsequent studies suggest intimidate relation between stress and the production of OMV on observing increase in OMV production during stress conditions (Beveridge et al., 1997; Baumgarten et al., 2012; Fulsundar et al., 2014).

### Secretion of Toxins, Adhesions, and Virulence Factors

Interaction of bacteria with host triggers release of OMVs, carrying proteins (OspA and OspB in *B. burgdorferi*) and other adhesion molecules (SabA, BabA, and VacA in *H. Pylori*, UspA1 in *M. catarrhalis* and aminopeptidase in *P. aeruginosa*) on their surface (MacDonald and Kuehn, 2012). OMVs acting as a bridge to increase adhesion of bacteria with the host tissues. They are also known to increase adherence of bacteria with epithelial linings of gut or the respiratory tract which helps bacteria resist their physical elimination. Association of OMVs with pathogen associated molecular patterns (PAMPs) such as LPS and porins, mediates strong immune response in endothelial cells and induces expression of pattern recognizing receptors on macrophages (Ellis and Kuehn, 2010; Kim et al., 2013). OMVs associated toxins such as leukotoxin, LPS, and ClyA are more potent than their soluble forms (Wai et al., 2003; Kuehn and Kesty, 2005). Secretion of shiga toxin from enterohemorrhagic *E. coli* O157:H7 and stx1 and stx2 of *S. dysenteriae* as cargo to OMVs efficiently inhibits eukaryotic protein synthesis (Yokoyama et al., 2000; Dutta et al., 2004). OMVs harboring multiple virulence factors such as Cif, PlcH, LPS, and alkaline phosphatase produce pronounced effect on host cells (Bomberger et al., 2009; Ellis and Kuehn, 2010). Secretion of toxins and virulence factors help bacteria to invade host cells, hijack host machinery for nutrient acquisition and also evade host defense system by modulating host immune response, which is pivotal for their fecundity and survival in the host.

### Invasion of Host and Modulation of Immune Defense

It is well established that OMVs have diverse biological functions i-e. from short range intercellular communications, to their long distance mode of action. They also perform immunomodulatory function by regulating delivery of different constituents to recipient cells (Corrado et al., 2013). Internalization of OMVs is achieved by four different pathways i-e. Macropinocytosis, Clathrin, Caveolin, and Lipid raft mediated endocytosis. Macropinocytosis is an actin driven process, which allows internalization of OMVs after sampling of extracellular medium at the cell surface. Macropinocytosis is reported from *S. flexneri*, where it helps to establish infection on host tissues (O’Donoghue and Krachler, 2016; Weiner et al., 2016). Clathrin mediated endocytosis via, clathrin coated pits occurs following ligand binding to cell surface receptor, triggering internalization of OVMs (Rewatkar et al., 2015). Unlike macropinocytosis that allows internalization of vesicles of 1 μm diameter, clathrin dependent endocytosis allows internalization of cargo, with a maximum of 120nm diameter. Clathrin mediated endocytosis is adapted for internalization of shiga toxin, CT, and cytotoxic virulence factor, VacA of *H. pylori* (Boisvert and Duncan, 2008; Parker et al., 2010). Caveolin mediated endocytosis occurs as cave shaped invagination (80nm) of membrane rich in cholesterol, sphingolipids, and caveolin. Despite sluggish internalization speed (five times less than clathrin mediated), it leads in the efficient delivery of cargo to the cytosol (Ritter et al., 1995; Mulcahy et al., 2014; Rewatkar et al., 2015). This type of transfer is utilized by *H. influenza*. Lipid raft (plasma membrane domains enriched in cholesterol and sphingolipids) mediated endocytosis is known for its involvement in the OMVs entry to host cells (Furuta et al., 2009; Jin et al., 2011; Sharpe et al., 2011; Mulcahy et al., 2014; Mondal et al., 2016). It is hypothesized that clustering of cholesterol rich regions causes curvature in membrane that drives movement of molecules as invagination (90nm) to host cell.

## Conclusion

Outer Membrane Vesicles are produced by a series of pathogenic and non-pathogenic bacteria. Devoid of cytosolic components, their cargo content (proteins and lipids) shows high similarity to the OM of the bacteria, from where they originate. Acting as a secretory system, molecules of definite composition destined to different cellular localizations are delivered in an active form, protected by membranous sheath. OMVs are associated with the release of molecules mediating bacterial survival in free state or in biofilm structures, thereby allowing maintenance of virulence, stress release, transformation along with adherence and colonization of bacteria to different hosts. Additionally, they play a significant role in bacterial adaptation by harboring enzymatic machinery for breakdown of complex material into nutrients and consequentially making it available for transportation into cells. With ability to shuttle molecules between cells; they facilitate cell-to-cell communication in a variety of biological processes. Their contribution in processes such as nutrient acquisition, intercellular communication, defense, and regulators of cellular niche has raised considerable interest of scientific fraternity for studying their role as a carrier in the delivery of therapeutics.

Outer Membrane Vesicles are speculated to modulate many physiological and pathological procedures. Exploiting their physiological characteristics, delivery of a series of therapeutic cargos (siRNA, microRNA, and proteins) to tissues has been already achieved (Raposo and Stoorvogel, 2013). Bioengineering aimed to achieve target specific delivery of therapeutics has also been exploited by designing potent liposomal nanocarriers. With a lipid bilayer topology, encapsulation of amphipathic therapeutics by liposomes, provides them a longer life span, besides increasing their stability with reduced side effects (Ozpolat et al., 2014). With maximum use in carrying drugs, liposomal encapsulated chemotherapeutic agents, such as doxorubicin shows its increased accumulation in tumor and significantly reduced toxicity, when compared with the use of free doxorubicin (Petros and DeSimone, 2010). The facilitation in the release of therapeutic cargo under specific conditions, calls for the bioengineering of liposomes based nanocarriers on the footprints of OMVs, to achieve better targeting and increased uptake of therapeutics.

## Author Contributions

AJ conceived the idea and contributed to writing of the manuscript.

## Conflict of Interest Statement

The author declares that the research was conducted in the absence of any commercial or financial relationships that could be construed as a potential conflict of interest.

## References

[B1] Alaniz R. C. Deatherage B. L. Lara J. C. Cookson B. T. (2007). Membra ne vesicles are immunogenic facsimiles of *Salmonella* typhimurium that potently activate dendritic cells, prime B and T cell responses, and stimulate protective immunity in vivo. *J. Immunol.* 179 7692–7701. 10.4049/jimmunol.179.11.7692 18025215

[B2] Allan N. D. Kooi C. Sokol P. A. Beveridge T. J. (2003). Putative virulence factors are released in association with membrane vesicles from *Burkholderia cepacia*. *Can. J. Microbiol.* 49 613–624. 10.1139/w03-078 14663495

[B3] Altindis E. Fu Y. Mekalanos J. J. (2014). Proteomic analysis of *Vibrio cholerae* outer membrane vesicles. *Proc. Natl. Acad. Sci. U.S.A.* 111 E1548–E1556. 10.1073/pnas.1403683111 24706774 PMC3992640

[B4] Bauman S. J. Kuehn M. J. (2006). Purification of outer membrane vesicles from *Pseudomonas aeruginosa* and their activation of an IL-8 response. *Microbes Infect.* 8 2400–2408. 10.1016/j.micinf.2006.05.001 16807039 PMC3525494

[B5] Baumgarten T. Sperling S. Seifert J. Von Bergen M. Steiniger F. Wick L. Y. (2012). Membrane vesicle formation as a multiple-stress response mechanism enhances *Pseudomonas putida* DOT-T1E cell surface hydrophobicity and biofilm formation. *Appl. Environ. Microbiol.* 78 6217–6224. 10.1128/AEM.01525-12 22752175 PMC3416621

[B6] Bergman M. A. Cummings L. A. Barrett S. L. Smith K. D. Lara J. C. Aderem A. (2005). CD4+ T cells and toll-like receptors recognize *Salmonella* antigens expressed in bacterial surface organelles. *Infect. Immun.* 73 1350–1356. 10.1128/IAI.73.3.1350-1356.2005 15731032 PMC1064935

[B7] Berleman J. E. Allen S. Danielewicz M. A. Remis J. P. Gorur A. Cunha J. (2014). The lethal cargo of *Myxococcus xanthus* outer membrane vesicles. *Front. Microbiol.* 5:474 10.3389/fmicb.2014.00474 PMC4158809 25250022

[B8] Bernadac A. Gavioli M. Lazzaroni J. C. Raina S. Lloubes R. (1998). *Escherichia coli* tol-pal mutants form outer membrane vesicles. *J. Bacteriol.* 180 4872–4878. 9733690 10.1128/jb.180.18.4872-4878.1998 PMC107512

[B9] Beveridge T. J. (1999). Structures of gram-negative cell walls and their derived membrane vesicles. *J. Bacteriol.* 181 4725–4733. 10438737 10.1128/jb.181.16.4725-4733.1999 PMC93954

[B10] Beveridge T. J. Makin S. A. Kadurugamuwa J. L. Li Z. (1997). Interactions between biofilms and the environment. *FEMS Microbiol. Rev.* 20 291–303. 10.1111/j.1574-6976.1997.tb00315.x 9299708

[B11] Biller S. J. Schubotz F. Roggensack S. E. Thompson A. W. Summons R. E. Chisholm S. W. (2014). Bacterial vesicles in marine ecosystems. *Science* 343 183–186. 10.1126/science.1243457 24408433

[B12] Bishop A. L. Schild S. Patimalla B. Klein B. Camilli A. (2010). Mucosal immunization with *Vibrio cholerae* outer membrane vesicles provides maternal protection mediated by antilipopolysaccharide antibodies that inhibit bacterial motility. *Infect. Immun.* 78 4402–4420. 10.1128/IAI.00398-10 20679439 PMC2950341

[B13] Boisvert H. Duncan M. J. (2008). Clathrin-dependent entry of a gingipain adhesin peptide and *Porphyromonas gingivalis* into host cells. *Cell Microbiol.* 10 2538–2552. 10.1111/j.1462-5822.2008.01228.x 18717820 PMC3016922

[B14] Bomberger J. M. Maceachran D. P. Coutermarsh B. A. Ye S. O’toole G. A. Stanton B. A. (2009). Long-distance delivery of bacterial virulence factors by *Pseudomonas aeruginosa* outer membrane vesicles. *PLoS Pathog.* 5:e1000382 10.1371/journal.ppat.1000382 PMC2661024 19360133

[B15] Bonnington K. E. Kuehn M. J. (2014). Protein selection and export via outer membrane vesicles. *Biochim. Biophys. Acta* 1843 1612–1619. 10.1016/j.bbamcr.2013.12.011 24370777 PMC4317292

[B16] Burdett I. D. Murray R. G. (1974). Electron microscope study of septum formation in *Escherichia coli* strains B and B-r during synchronous growth. *J. Bacteriol.* 119 1039–1056. 4604418 10.1128/jb.119.3.1039-1056.1974 PMC245711

[B17] Cascales E. Bernadac A. Gavioli M. Lazzaroni J. C. Lloubes R. (2002). Pal lipoprotein of *Escherichia coli* plays a major role in outer membrane integrity. *J. Bacteriol.* 184 754–759. 10.1128/JB.184.3.754-759.2002 11790745 PMC139529

[B18] Chatterjee D. Chaudhuri K. (2011). Association of cholera toxin with *Vibrio cholerae* outer membrane vesicles which are internalized by human intestinal epithelial cells. *FEBS Lett.* 585 1357–1362. 10.1016/j.febslet.2011.04.017 21510946

[B19] Chattopadhyay M. K. Jaganandham M. V. (2015). Vesicles-mediated resistance to antibiotics in bacteria. *Front. Microbiol.* 6:758 10.3389/fmicb.2015.00758 PMC4511839 26257725

[B20] Chi B. Qi M. Kuramitsu H. K. (2003). Role of dentilisin in *Treponema denticola* epithelial cell layer penetration. *Res. Microbiol.* 154 637–643. 10.1016/j.resmic.2003.08.001 14596901

[B21] Choi D. S. Kim D. K. Choi S. J. Lee J. Choi J. P. Rho S. (2011). Proteomic analysis of outer membrane vesicles derived from *Pseudomonas aeruginosa*. *Proteomics* 11 3424–3429. 10.1002/pmic.201000212 21751344

[B22] Chowdhury C. Jagannadham M. V. (2013). Virulence factors are released in association with outer membrane vesicles of *Pseudomonas* syringae pv. *tomato* T1 during normal growth. *Biochim. Biophys. Acta* 1834 231–239. 10.1016/j.bbapap.2012.09.015 23043909

[B23] Ciofu O. Beveridge T. J. Kadurugamuwa J. Walther-Rasmussen J. Hoiby N. (2000). Chromosomal beta-lactamase is packaged into membrane vesicles and secreted from *Pseudomonas aeruginosa*. *J. Antimicrob. Chemother.* 45 9–13. 10.1093/jac/45.1.9 10629007

[B24] Collins B. S. (2011). Gram-negative outer membrane vesicles in vaccine development. *Discov. Med.* 12 7–15. 21794204

[B25] Corrado C. Raimondo S. Chiesi A. Ciccia F. De Leo G. Alessandro R. (2013). Exosomes as intercellular signaling organelles involved in health and disease: basic science and clinical applications. *Int. J. Mol. Sci.* 14 5338–5366. 10.3390/ijms14035338 23466882 PMC3634447

[B26] Devoe I. W. Gilchrist J. E. (1973). Release of endotoxin in the form of cell wall blebs during in vitro growth of *Neisseria meningitidis*. *J. Exp. Med.* 138 1156–1167. 10.1084/jem.138.5.1156 4200775 PMC2139435

[B27] Dorward D. W. Garon C. F. (1989). DNA-binding proteins in cells and membrane blebs of *Neisseria gonorrhoeae*. *J. Bacteriol.* 171 4196–4201. 10.1128/jb.171.8.4196-4201.1989 2502535 PMC210190

[B28] Dorward D. W. Schwan T. G. Garon C. F. (1991). Immune capture and detection of *Borrelia burgdorferi* antigens in urine, blood, or tissues from infected ticks, mice, dogs, and humans. *J. Clin. Microbiol.* 29 1162–1170. 1864935 10.1128/jcm.29.6.1162-1170.1991 PMC269963

[B29] Dutta S. Iida K. Takade A. Meno Y. Nair G. B. Yoshida S. (2004). Release of Shiga toxin by membrane vesicles in *Shigella dysenteriae* serotype 1 strains and in vitro effects of antimicrobials on toxin production and release. *Microbiol. Immunol.* 48 965–969. 10.1111/j.1348-0421.2004.tb03626.x 15611613

[B30] Eddy J. L. Gielda L. M. Caulfield A. J. Rangel S. M. Lathem W. W. (2014). Production of outer membrane vesicles by the plague pathogen *Yersinia pestis*. *PLoS ONE* 9:e107002 10.1371/journal.pone.0107002 PMC4157834 25198697

[B31] Eggert U. S. Ruiz N. Falcone B. V. Branstrom A. A. Goldman R. C. Silhavy T. J. (2001). Genetic basis for activity differences between vancomycin and glycolipid derivatives of vancomycin. *Science* 294 361–364. 10.1126/science.1063611 11520949

[B32] Elhenawy W. Bording-Jorgensen M. Valguarnera E. Haurat M. F. Wine E. Feldman M. F. (2016). LPS remodeling triggers formation of outer membrane vesicles in *Salmonella*. *MBio* 7:e00940–16 10.1128/mBio.00940-16 PMC4958258 27406567

[B33] Ellis T. N. Kuehn M. J. (2010). Virulence and immunomodulatory roles of bacterial outer membrane vesicles. *Microbiol. Mol. Biol. Rev.* 74 81–94. 10.1128/MMBR.00031-09 20197500 PMC2832350

[B34] Ellis T. N. Leiman S. A. Kuehn M. J. (2010). Naturally produced outer membrane vesicles from *Pseudomonas aeruginosa* elicit a potent innate immune response via combined sensing of both lipopolysaccharide and protein components. *Infect. Immun.* 78 3822–3831. 10.1128/IAI.00433-10 20605984 PMC2937433

[B35] Elmi A. Watson E. Sandu P. Gundogdu O. Mills D. C. Inglis N. F. (2012). *Campylobacter jejuni* outer membrane vesicles play an important role in bacterial interactions with human intestinal epithelial cells. *Infect. Immun.* 80 4089–4098. 10.1128/IAI.00161-12 22966047 PMC3497446

[B36] Evans A. G. Davey H. M. Cookson A. Currinn H. Cooke-Fox G. Stanczyk P. J. (2012). Predatory activity of *Myxococcus xanthus* outer-membrane vesicles and properties of their hydrolase cargo. *Microbiology* 158 2742–2752. 10.1099/mic.0.060343-0 22977088

[B37] Fernandez-Moreira E. Helbig J. H. Swanson M. S. (2006). Membrane vesicles shed by *Legionella pneumophila* inhibit fusion of phagosomes with lysosomes. *Infect. Immun.* 74 3285–3295. 10.1128/IAI.01382-05 16714556 PMC1479291

[B38] Ferrari G. Garaguso I. Adu-Bobie J. Doro F. Taddei A. R. Biolchi A. (2006). Outer membrane vesicles from group B *Neisseria meningitidis* delta gna33 mutant: proteomic and immunological comparison with detergent-derived outer membrane vesicles. *Proteomics* 6 1856–1866. 10.1002/pmic.200500164 16456881

[B39] Fiocca R. Necchi V. Sommi P. Ricci V. Telford J. Cover T. L. (1999). Release of *Helicobacter pylori* vacuolating cytotoxin by both a specific secretion pathway and budding of outer membrane vesicles. Uptake of released toxin and vesicles by gastric epithelium. *J. Pathol.* 188 220–226. 10.1002/(SICI)1096-9896(199906)188:2<220::AID-PATH307>3.0.CO;2-C 10398168

[B40] Fulsundar S. Harms K. Flaten G. E. Johnsen P. J. Chopade B. A. Nielsen K. M. (2014). Gene transfer potential of outer membrane vesicles of *Acinetobacter baylyi* and effects of stress on vesiculation. *Appl. Environ. Microbiol.* 80 3469–3483. 10.1128/AEM.04248-13 24657872 PMC4018862

[B41] Furuta N. Takeuchi H. Amano A. (2009). Entry of *Porphyromonas gingivalis* outer membrane vesicles into epithelial cells causes cellular functional impairment. *Infect. Immun.* 77 4761–4770. 10.1128/IAI.00841-09 19737899 PMC2772519

[B42] Galka F. Wai S. N. Kusch H. Engelmann S. Hecker M. Schmeck B. (2008). Proteomic characterization of the whole secretome of *Legionella pneumophila* and functional analysis of outer membrane vesicles. *Infect. Immun.* 76 1825–1836. 10.1128/IAI.01396-07 18250176 PMC2346698

[B43] Haurat M. F. Aduse-Opoku J. Rangarajan M. Dorobantu L. Gray M. R. Curtis M. A. (2011). Selective sorting of cargo proteins into bacterial membrane vesicles. *J. Biol. Chem.* 286 1269–1276. 10.1074/jbc.M110.185744 21056982 PMC3020734

[B44] Haurat M. F. Elhenawy W. Feldman M. F. (2015). Prokaryotic membrane vesicles: new insights on biogenesis and biological roles. *Biol. Chem.* 396 95–109. 10.1515/hsz-2014-0183 25178905

[B45] Hoekstra D. Van Der Laan J. W. De Leij L. Witholt B. (1976). Release of outer membrane fragments from normally growing *Escherichia coli*. *Biochim. Biophys. Acta* 455 889–899. 10.1016/0005-2736(76)90058-4 793634

[B46] Horstman A. L. Kuehn M. J. (2000). Enterotoxigenic *Escherichia coli* secretes active heat-labile enterotoxin via outer membrane vesicles. *J. Biol. Chem.* 275 12489–12496. 10.1074/jbc.275.17.12489 10777535 PMC4347834

[B47] Hozbor D. Rodriguez M. E. Fernandez J. Lagares A. Guiso N. Yantorno O. (1999). Release of outer membrane vesicles from *Bordetella pertussis*. *Curr. Microbiol.* 38 273–278. 10.1007/PL00006801 10355115

[B48] Jang K. S. Sweredoski M. J. Graham R. L. Hess S. Clemons W. M. Jr. (2014). Comprehensive proteomic profiling of outer membrane vesicles from *Campylobacter jejuni*. *J. Proteomics* 98 90–98. 10.1016/j.jprot.2013.12.014 24382552 PMC4534003

[B49] Jin J. S. Kwon S. O. Moon D. C. Gurung M. Lee J. H. Kim S. I. (2011). *Acinetobacter baumannii* secretes cytotoxic outer membrane protein A via outer membrane vesicles. *PLoS ONE* 6:e17027 10.1371/journal.pone.0017027 PMC3046175 21386968

[B50] Jun S. H. Lee J. H. Kim B. R. Kim S. I. Park T. I. Lee J. C. (2013). *Acinetobacter baumannii* outer membrane vesicles elicit a potent innate immune response via membrane proteins. *PLoS ONE* 8:e71751 10.1371/journal.pone.0071751 PMC3743744 23977136

[B51] Kadurugamuwa J. L. Beveridge T. J. (1995). Virulence factors are released from *Pseudomonas aeruginosa* in association with membrane vesicles during normal growth and exposure to gentamicin: a novel mechanism of enzyme secretion. *J. Bacteriol.* 177 3998–4008. 10.1128/jb.177.14.3998-4008.1995 7608073 PMC177130

[B52] Kadurugamuwa J. L. Beveridge T. J. (1996). Bacteriolytic effect of membrane vesicles from *Pseudomonas aeruginosa* on other bacteria including pathogens: conceptually new antibiotics. *J. Bacteriol.* 178 2767–2774. 10.1128/jb.178.10.2767-2774.1996 8631663 PMC178010

[B53] Kadurugamuwa J. L. Beveridge T. J. (1998). Delivery of the non-membrane-permeative antibiotic gentamicin into mammalian cells by using *Shigella flexneri* membrane vesicles. *Antimicrob. Agents Chemother.* 42 1476–1483. 9624497 10.1128/aac.42.6.1476 PMC105625

[B54] Kadurugamuwa J. L. Beveridge T. J. (1999). Membrane vesicles derived from *Pseudomonas aeruginosa* and *Shigella flexneri* can be integrated into the surfaces of other gram-negative bacteria. *Microbiology* 145(Pt 8), 2051–2060. 10.1099/13500872-145-8-2051 10463171

[B55] Kato S. Kowashi Y. Demuth D. R. (2002). Outer membrane-like vesicles secreted by *Actinobacillus actinomycetemcomitans* are enriched in leukotoxin. *Microb. Pathog.* 32 1–13. 10.1006/mpat.2001.0474 11782116

[B56] Keenan J. Day T. Neal S. Cook B. Perez-Perez G. Allardyce R. (2000). A role for the bacterial outer membrane in the pathogenesis of *Helicobacter* pylori infection. *FEMS Microbiol. Lett.* 182 259–264. 10.1111/j.1574-6968.2000.tb08905.x 10620676

[B57] Kim Y. S. Choi E. J. Lee W. H. Choi S. J. Roh T. Y. Park J. (2013). Extracellular vesicles, especially derived from Gram-negative bacteria, in indoor dust induce neutrophilic pulmonary inflammation associated with both Th1 and Th17 cell responses. *Clin. Exp. Allergy* 43 443–454. 10.1111/cea.12085 23517040

[B58] Klimentova J. Stulik J. (2015). Methods of isolation and purification of outer membrane vesicles from gram-negative bacteria. *Microbiol. Res.* 170 1–9. 10.1016/j.micres.2014.09.006 25458555

[B59] Koeppen K. Hampton T. H. Jarek M. Scharfe M. Gerber S. A. Mielcarz D. W. (2016). A novel mechanism of host-pathogen interaction through sRNA in bacterial outer membrane vesicles. *PLoS Pathog.* 12:e1005672 10.1371/journal.ppat.1005672 PMC4905634 27295279

[B60] Kolling G. L. Matthews K. R. (1999). Export of virulence genes and Shiga toxin by membrane vesicles of *Escherichia coli* O157:H7. *Appl. Environ. Microbiol.* 65 1843–1848. 10223967 10.1128/aem.65.5.1843-1848.1999 PMC91264

[B61] Kothary M. H. Gopinath G. R. Gangiredla J. Rallabhandi P. V. Harrison L. M. Yan Q. Q. (2017). Analysis and characterization of proteins associated with outer membrane vesicles secreted by *Cronobacter* spp. *Front. Microbiol.* 8:134 10.3389/fmicb.2017.00134 PMC5299011 28232819

[B62] Kuehn M. J. Kesty N. C. (2005). Bacterial outer membrane vesicles and the host-pathogen interaction. *Genes Dev.* 19 2645–2655. 10.1101/gad.1299905 16291643

[B63] Kulkarni H. M. Jagannadham M. V. (2014). Biogenesis and multifaceted roles of outer membrane vesicles from Gram-negative bacteria. *Microbiology* 160 2109–2121. 10.1099/mic.0.079400-0 25069453

[B64] Kulp A. Kuehn M. J. (2010). Biological functions and biogenesis of secreted bacterial outer membrane vesicles. *Annu. Rev. Microbiol.* 64 163–184. 10.1146/annurev.micro.091208.073413 20825345 PMC3525469

[B65] Kwon S. O. Gho Y. S. Lee J. C. Kim S. I. (2009). Proteome analysis of outer membrane vesicles from a clinical *Acinetobacter baumannii* isolate. *FEMS Microbiol. Lett.* 297 150–156. 10.1111/j.1574-6968.2009.01669.x 19548894

[B66] Lee E. Y. Choi D. S. Kim K. P. Gho Y. S. (2008). Proteomics in gram-negative bacterial outer membrane vesicles. *Mass Spectrom. Rev.* 27 535–555. 10.1002/mas.20175 18421767

[B67] Lee J. Kim O. Y. Gho Y. S. (2016). Proteomic profiling of Gram-negative bacterial outer membrane vesicles: current perspectives. *Proteomics Clin. Appl.* 10 897–909. 10.1002/prca.201600032 27480505

[B68] Li Z. Clarke A. J. Beveridge T. J. (1998). Gram-negative bacteria produce membrane vesicles which are capable of killing other bacteria. *J. Bacteriol.* 180 5478–5483. 9765585 10.1128/jb.180.20.5478-5483.1998 PMC107602

[B69] Lindmark B. Rompikuntal P. K. Vaitkevicius K. Song T. Mizunoe Y. Uhlin B. E. (2009). Outer membrane vesicle-mediated release of cytolethal distending toxin (CDT) from *Campylobacter jejuni*. *BMC Microbiol.* 9:220 10.1186/1471-2180-9-220 PMC2770062 19835618

[B70] MacDonald I. A. Kuehn M. J. (2012). Offense and defense: microbial membrane vesicles play both ways. *Res. Microbiol.* 163 607–618. 10.1016/j.resmic.2012.10.020 23123555 PMC3518640

[B71] Manning A. J. Kuehn M. J. (2011). Contribution of bacterial outer membrane vesicles to innate bacterial defense. *BMC Microbiol.* 11:258 10.1186/1471-2180-11-258 PMC3248377 22133164

[B72] Manning A. J. Kuehn M. J. (2013). Functional advantages conferred by extracellular prokaryotic membrane vesicles. *J. Mol. Microbiol. Biotechnol.* 23 131–141. 10.1159/000346548 23615201 PMC4324172

[B73] Mashburn L. M. Whiteley M. (2005). Membrane vesicles traffic signals and facilitate group activities in a prokaryote. *Nature* 437 422–425. 10.1038/nature03925 16163359

[B74] Mashburn-Warren L. Howe J. Brandenburg K. Whiteley M. (2009). Structural requirements of the *Pseudomonas* quinolone signal for membrane vesicle stimulation. *J. Bacteriol.* 191 3411–3414. 10.1128/JB.00052-09 19286801 PMC2687154

[B75] Mashburn-Warren L. Howe J. Garidel P. Richter W. Steiniger F. Roessle M. (2008). Interaction of quorum signals with outer membrane lipids: insights into prokaryotic membrane vesicle formation. *Mol. Microbiol.* 69 491–502. 10.1111/j.1365-2958.2008.06302.x 18630345 PMC2615190

[B76] Mashburn-Warren L. M. Whiteley M. (2006). Special delivery: vesicle trafficking in prokaryotes. *Mol. Microbiol.* 61 839–846. 10.1111/j.1365-2958.2006.05272.x 16879642

[B77] Massari P. Gunawardana J. Liu X. Wetzler L. M. (2010). Meningococcal porin PorB prevents cellular apoptosis in a toll-like receptor 2- and NF-kappaB-independent manner. *Infect. Immun.* 78 994–1003. 10.1128/IAI.00156-09 20028813 PMC2825956

[B78] McBroom A. J. Kuehn M. J. (2007). Release of outer membrane vesicles by Gram-negative bacteria is a novel envelope stress response. *Mol. Microbiol.* 63 545–558. 10.1111/j.1365-2958.2006.05522.x 17163978 PMC1868505

[B79] McCaig W. D. Koller A. Thanassi D. G. (2013). Production of outer membrane vesicles and outer membrane tubes by *Francisella novicida*. *J. Bacteriol.* 195 1120–1132. 10.1128/JB.02007-12 23264574 PMC3592013

[B80] McMahon K. J. Castelli M. E. Garcia Vescovi E. Feldman M. F. (2012). Biogenesis of outer membrane vesicles in *Serratia marcescens* is thermoregulated and can be induced by activation of the Rcs phosphorelay system. *J. Bacteriol.* 194 3241–3249. 10.1128/JB.00016-12 22493021 PMC3370869

[B81] Mendez J. A. Soares N. C. Mateos J. Gayoso C. Rumbo C. Aranda J. (2012). Extracellular proteome of a highly invasive multidrug-resistant clinical strain of *Acinetobacter baumannii*. *J. Proteome Res.* 11 5678–5694. 10.1021/pr300496c 22966805

[B82] Mondal A. Tapader R. Chatterjee N. S. Ghosh A. Sinha R. Koley H. (2016). Cytotoxic and inflammatory responses induced by outer membrane vesicle-associated biologically active proteases from *Vibrio cholerae*. *Infect. Immun.* 84 1478–1490. 10.1128/IAI.01365-15 26930702 PMC4862697

[B83] Moon D. C. Choi C. H. Lee J. H. Choi C. W. Kim H. Y. Park J. S. (2012). *Acinetobacter baumannii* outer membrane protein A modulates the biogenesis of outer membrane vesicles. *J. Microbiol* 50 155–160. 10.1007/s12275-012-1589-4 22367951

[B84] Mulcahy L. A. Pink R. C. Carter D. R. (2014). Routes and mechanisms of extracellular vesicle uptake. *J. Extracell Vesicles* 3 24641 10.3402/jev.v3.24641 PMC4122821 25143819

[B85] Mullaney E. Brown P. A. Smith S. M. Botting C. H. Yamaoka Y. Y. Terres A. M. (2009). Proteomic and functional characterization of the outer membrane vesicles from the gastric pathogen *Helicobacter pylori*. *Proteomics Clin. Appl.* 3 785–796. 10.1002/prca.200800192 21136987

[B86] Namork E. Brandtzaeg P. (2002). Fatal meningococcal septicaemia with “blebbing” meningococcus. *Lancet* 360 1741 10.1016/S0140-6736(02)11721-1 12480427

[B87] O’Donoghue E. J. Krachler A. M. (2016). Mechanisms of outer membrane vesicle entry into host cells. *Cell Microbiol.* 18 1508–1517. 10.1111/cmi.12655 27529760 PMC5091637

[B88] Olofsson A. Vallstrom A. Petzold K. Tegtmeyer N. Schleucher J. Carlsson S. (2010). Biochemical and functional characterization of *Helicobacter pylori* vesicles. *Mol. Microbiol.* 77 1539–1555. 10.1111/j.1365-2958.2010.07307.x 20659286 PMC3068288

[B89] Ozpolat B. Sood A. K. Lopez-Berestein G. (2014). Liposomal siRNA nanocarriers for cancer therapy. *Adv. Drug Deliv. Rev.* 66 110–116. 10.1016/j.addr.2013.12.008 24384374 PMC4527165

[B90] Parker H. Chitcholtan K. Hampton M. B. Keenan J. I. (2010). Uptake of *Helicobacter pylori* outer membrane vesicles by gastric epithelial cells. *Infect. Immun.* 78 5054–5061. 10.1128/IAI.00299-10 20876296 PMC2981328

[B91] Petros R. A. DeSimone J. M. (2010). Strategies in the design of nanoparticles for therapeutic applications. *Nat. Rev. Drug Discov.* 9 615–627. 10.1038/nrd2591 20616808

[B92] Pettit R. K. Judd R. C. (1992). The interaction of naturally elaborated blebs from serum-susceptible and serum-resistant strains of *Neisseria gonorrhoeae* with normal human serum. *Mol. Microbiol.* 6 729–734. 10.1111/j.1365-2958.1992.tb01522.x 1574002

[B93] Raposo G. Stoorvogel W. (2013). Extracellular vesicles: exosomes, microvesicles, and friends. *J. Cell Biol.* 200 373–383. 10.1083/jcb.201211138 23420871 PMC3575529

[B94] Raymond B. Bonsall M. B. (2013). Cooperation and the evolutionary ecology of bacterial virulence: the *Bacillus cereus* group as a novel study system. *Bioessays* 35 706–716. 10.1002/bies.201300028 23702950

[B95] Renelli M. Matias V. Lo R. Y. Beveridge T. J. (2004). DNA-containing membrane vesicles of *Pseudomonas aeruginosa* PAO1 and their genetic transformation potential. *Microbiology* 150 2161–2169. 10.1099/mic.0.26841-0 15256559

[B96] Rewatkar P. V. Parton R. G. Parekh H. S. Parat M. O. (2015). Are caveolae a cellular entry route for non-viral therapeutic delivery systems? *Adv. Drug Deliv. Rev.* 91 92–108. 10.1016/j.addr.2015.01.003 25579057

[B97] Ritter T. E. Fajardo O. Matsue H. Anderson R. G. Lacey S. W. (1995). Folate receptors targeted to clathrin-coated pits cannot regulate vitamin uptake. *Proc. Natl. Acad. Sci. U.S.A.* 92 3824–3828. 10.1073/pnas.92.9.3824 7731991 PMC42054

[B98] Rolhion N. Barnich N. Claret L. Darfeuille-Michaud A. (2005). Strong decrease in invasive ability and outer membrane vesicle release in Crohn’s disease-associated adherent-invasive *Escherichia coli* strain LF82 with the yfgL gene deleted. *J. Bacteriol.* 187 2286–2296. 10.1128/JB.187.7.2286-2296.2005 15774871 PMC1065249

[B99] Rosen G. Naor R. Rahamim E. Yishai R. Sela M. N. (1995). Proteases of *Treponema denticola* outer sheath and extracellular vesicles. *Infect. Immun.* 63 3973–3979. 7558307 10.1128/iai.63.10.3973-3979.1995 PMC173558

[B100] Schertzer J. W. Whiteley M. (2012). A bilayer-couple model of bacterial outer membrane vesicle biogenesis. *MBio* 3:e00297–11 10.1128/mBio.00297-11 PMC3312216 22415005

[B101] Schooling S. R. Beveridge T. J. (2006). Membrane vesicles: an overlooked component of the matrices of biofilms. *J. Bacteriol.* 188 5945–5957. 10.1128/JB.00257-06 16885463 PMC1540058

[B102] Schwechheimer C. Kuehn M. J. (2015). Outer-membrane vesicles from Gram-negative bacteria: biogenesis and functions. *Nat. Rev. Microbiol.* 13 605–619. 10.1038/nrmicro3525 26373371 PMC5308417

[B103] Schwechheimer C. Sullivan C. J. Kuehn M. J. (2013). Envelope control of outer membrane vesicle production in Gram-negative bacteria. *Biochemistry* 52 3031–3040. 10.1021/bi400164t 23521754 PMC3731998

[B104] Sharpe S. W. Kuehn M. J. Mason K. M. (2011). Elicitation of epithelial cell-derived immune effectors by outer membrane vesicles of nontypeable *Haemophilus influenzae*. *Infect. Immun.* 79 4361–4369. 10.1128/IAI.05332-11 21875967 PMC3257905

[B105] Shoberg R. J. Thomas D. D. (1993). Specific adherence of *Borrelia burgdorferi* extracellular vesicles to human endothelial cells in culture. *Infect. Immun.* 61 3892–3900. 8359911 10.1128/iai.61.9.3892-3900.1993 PMC281091

[B106] Shoberg R. J. Thomas D. D. (1995). *Borrelia burgdorferi* vesicle production occurs via a mechanism independent of immunoglobulin M involvement. *Infect. Immun.* 63 4857–4861. 7591146 10.1128/iai.63.12.4857-4861.1995 PMC173695

[B107] Sidhu V. K. Vorholter F. J. Niehaus K. Watt S. A. (2008). Analysis of outer membrane vesicle associated proteins isolated from the plant pathogenic bacterium *Xanthomonas campestris* pv. *campestris*. *BMC Microbiol.* 8:87 10.1186/1471-2180-8-87 PMC2438364 18518965

[B108] Spitzer J. Poolman B. (2013). How crowded is the prokaryotic cytoplasm? *FEBS Lett.* 587 2094–2098. 10.1016/j.febslet.2013.05.051 23735698

[B109] Tan T. T. Morgelin M. Forsgren A. Riesbeck K. (2007). *Haemophilus influenzae* survival during complement-mediated attacks is promoted by *Moraxella catarrhalis* outer membrane vesicles. *J. Infect. Dis.* 195 1661–1670. 10.1086/517611 17471436

[B110] Tashiro Y. Ichikawa S. Shimizu M. Toyofuku M. Takaya N. Nakajima-Kambe T. (2010). Variation of physiochemical properties and cell association activity of membrane vesicles with growth phase in *Pseudomonas aeruginosa*. *Appl. Environ. Microbiol.* 76 3732–3739. 10.1128/AEM.02794-09 20382806 PMC2876431

[B111] Tashiro Y. Inagaki A. Shimizu M. Ichikawa S. Takaya N. Nakajima-Kambe T. (2011). Characterization of phospholipids in membrane vesicles derived from *Pseudomonas aeruginosa*. *Biosci. Biotechnol. Biochem.* 75 605–607. 10.1271/bbb.100754 21389607

[B112] Toledo A. Coleman J. L. Kuhlow C. J. Crowley J. T. Benach J. L. (2012). The enolase of *Borrelia burgdorferi* is a plasminogen receptor released in outer membrane vesicles. *Infect. Immun.* 80 359–368. 10.1128/IAI.05836-11 22083700 PMC3255694

[B113] Toyofuku M. Roschitzki B. Riedel K. Eberl L. (2012). Identification of proteins associated with the *Pseudomonas aeruginosa* biofilm extracellular matrix. *J. Proteome Res.* 11 4906–4915. 10.1021/pr300395j 22909304

[B114] Turner L. Praszkier J. Hutton M. L. Steer D. Ramm G. Kaparakis-Liaskos M. (2015). Increased outer membrane vesicle formation in a *Helicobacter pylori* tolB mutant. *Helicobacter* 20 269–283. 10.1111/hel.12196 25669590

[B115] Unal C. M. Schaar V. Riesbeck K. (2011). Bacterial outer membrane vesicles in disease and preventive medicine. *Semin. Immunopathol.* 33 395–408. 10.1007/s00281-010-0231-y 21153593

[B116] Van De Waterbeemd B. Mommen G. P. Pennings J. L. Eppink M. H. Wijffels R. H. Van Der Pol L. A. (2013). Quantitative proteomics reveals distinct differences in the protein content of outer membrane vesicle vaccines. *J. Proteome Res.* 12 1898–1908. 10.1021/pr301208g 23410224

[B117] Vanaja S. K. Russo A. J. Behl B. Banerjee I. Yankova M. Deshmukh S. D. (2016). Bacterial outer membrane vesicles mediate cytosolic localization of LPS and caspase-11 activation. *Cell* 165 1106–1119. 10.1016/j.cell.2016.04.015 27156449 PMC4874922

[B118] Vasilyeva N. V. Tsfasman I. M. Suzina N. E. Stepnaya O. A. Kulaev I. S. (2008). Secretion of bacteriolytic endopeptidase L5 of *Lysobacter* sp. XL1 into the medium by means of outer membrane vesicles. *FEBS J.* 275 3827–3835. 10.1111/j.1742-4658.2008.06530.x 18573103

[B119] Veith P. D. Chen Y. Y. Gorasia D. G. Chen D. Glew M. D. O’brien-Simpson N. M. (2014). *Porphyromonas gingivalis* outer membrane vesicles exclusively contain outer membrane and periplasmic proteins and carry a cargo enriched with virulence factors. *J. Proteome Res.* 13 2420–2432. 10.1021/pr401227e 24620993

[B120] Vidakovics M. L. Jendholm J. Morgelin M. Mansson A. Larsson C. Cardell L. O. (2010). B cell activation by outer membrane vesicles–a novel virulence mechanism. *PLoS Pathog.* 6:e1000724 10.1371/journal.ppat.1000724 PMC2799554 20090836

[B121] Vipond C. Suker J. Jones C. Tang C. Feavers I. M. Wheeler J. X. (2006). Proteomic analysis of a meningococcal outer membrane vesicle vaccine prepared from the group B strain NZ98/254. *Proteomics* 6 3400–3413. 10.1002/pmic.200500821 16645985

[B122] Wai S. N. Lindmark B. Soderblom T. Takade A. Westermark M. Oscarsson J. (2003). Vesicle-mediated export and assembly of pore-forming oligomers of the enterobacterial ClyA cytotoxin. *Cell* 115 25–35. 10.1016/S0092-8674(03)00754-2 14532000

[B123] Weiner A. Mellouk N. Lopez-Montero N. Chang Y. Y. Souque C. Schmitt C. (2016). Macropinosomes are key players in early *Shigella* invasion and vacuolar escape in epithelial cells. *PLoS Pathog.* 12:e1005602 10.1371/journal.ppat.1005602 PMC4868309 27182929

[B124] Wensink J. Witholt B. (1981). Outer-membrane vesicles released by normally growing *Escherichia coli* contain very little lipoprotein. *Eur. J. Biochem.* 116 331–335. 10.1111/j.1432-1033.1981.tb05338.x 7018907

[B125] Yaron S. Kolling G. L. Simon L. Matthews K. R. (2000). Vesicle-mediated transfer of virulence genes from *Escherichia coli* O157:H7 to other enteric bacteria. *Appl. Environ. Microbiol.* 66 4414–4420. 10.1128/AEM.66.10.4414-4420.2000 11010892 PMC92318

[B126] Yokoyama K. Horii T. Yamashino T. Hashikawa S. Barua S. Hasegawa T. (2000). Production of shiga toxin by *Escherichia coli* measured with reference to the membrane vesicle-associated toxins. *FEMS Microbiol. Lett.* 192 139–144. 10.1111/j.1574-6968.2000.tb09372.x 11040442

